# Genetic Insights Into Early-Onset Type 2 Diabetes Mellitus: The Role of SOST and LRP5 Genotypic Variants in Young Indians

**DOI:** 10.7759/cureus.80186

**Published:** 2025-03-06

**Authors:** Jiya Singh, Praveen K Singh, Rahul Amoli, Ravi Kant, Anissa A Mirza, Manisha Naithani, Sarama Saha

**Affiliations:** 1 Biochemistry, All India Institute of Medical Sciences, Rishikesh, Rishikesh, IND; 2 Biochemistry, Maharishi Markandeshwar Medical College & Hospital, Solan, IND; 3 General Medicine, All India Institute of Medical Sciences, Rishikesh, Rishikesh, IND

**Keywords:** early-onset diabetes, gene polymorphism, low-density lipoprotein receptor-related protein-5 (lrp5), sclerostin (sost), type 2 diabetes mellitus (t2dm), wnt signaling pathway

## Abstract

Background

Type 2 diabetes mellitus (T2DM) presents a significant global health challenge, with increasing prevalence rates. The pathogenesis of early-onset T2DM is complex, with the Wnt signaling pathway playing a crucial role in islet cell development.

Purpose

This study was designed to explore the association of sclerostin (*SOST*) and low-density lipoprotein receptor-related protein-5 (*LRP5*) gene polymorphisms with early-onset T2DM in the young population of Uttarakhand, which is novel given that no studies have focused on this link within an Indian demographic.

Methodology

In this case-control study, T2DM patients between 20 and 40 years old who attended Medicine OPD were recruited as cases. After taking informed consent, 5 mL of blood was collected. Serum was used for lipid profile using the enzymatic method and sclerostin using the enzyme-linked immunosorbent assay (ELISA) method. DNA isolated from whole blood was subjected to polymerase chain reaction-restriction fragment length polymorphism (PCR-RFLP) for restriction digestion by NCoI and HinfI enzymes. Statistical analysis was done using IBM SPSS Statistics for Windows, Version 23 (Released 2015; IBM Corp., Armonk, New York). A comparison of genotypic variants was made using a chi-square test.

Results

In this study of 114 early-onset T2DM cases and 115 sex-matched controls, genotype distributions of the gene that codes for the protein sclerostin (*SOST*) rs865429 and *LRP5* rs11228303 followed Hardy-Weinberg equilibrium. In cases, the *SOST* GG genotype showed a nonsignificant higher odds ratio (OR=2.035) for T2DM, and it was associated with higher low-density lipoprotein (LDL) levels (p=0.027), HbA1c, and triglycerides. The *LRP5* CT genotype in cases was linked to significantly higher HbA1c (p=0.018) and elevated triglycerides and calcium, while the TT genotype showed the highest levels across multiple clinical parameters. The *SOST* GG genotype also suggested a possible link with bone metabolism due to elevated calcium, intact parathyroid hormone (iPTH), and sclerostin levels. These findings indicate potential interactions between *SOST* and *LRP5* variants and metabolic markers in early-onset T2DM.

Conclusion

The trend of elevated biochemical parameters in GG and TT genotypes and higher odds ratio (OR=2.035) concludes that *SOST* and *LRP5* variants may contribute to early-onset T2DM and related complications. These findings highlight the importance of genetic predisposition in T2DM, pointing to a need for personalized clinical approaches. Larger, multicenter studies are needed to confirm these preliminary results.

## Introduction

Type 2 diabetes mellitus (T2DM) is a global health issue. According to Wang et al., the International Diabetes Federation (IDF) Diabetes Atlas 2021 estimates that 537.5 million adults worldwide were living with diabetes in 2021, with projections indicating a rise to 783 million by 2045 [1]. Kumar et al. documented that the prevalence of diabetes has escalated to 9.6% in 2021, affecting around 135 million individuals [2]. Surprisingly, approximately 57% of these cases remain undiagnosed. The cause of early-onset T2DM is complex and multifactorial. Recent studies are increasingly focused on identifying specific genetic polymorphisms that might be implicated in the risk and development of early-onset T2DM.

The Wingless-related integration site (Wnt) signaling pathway plays a crucial role in the regulation of various cellular processes such as proliferation, differentiation, and apoptosis. Sclerostin, a Wnt signaling pathway inhibitor, was found to be significantly increased in prediabetic and diabetic individuals compared to healthy individuals [3]. Moreover, glycoxidatively modified lipoproteins were observed to contribute to diabetic complications via modulation of aldosterone release [4,5]. Low-density lipoprotein receptor-related protein 5 (LRP5) plays a critical role in bone, glucose, and cholesterol metabolism through its involvement in the Wnt/β-catenin signaling pathway [6], implying a potential link between *LRP5* gene variants and T2DM. However, based on the literature search, no study has been conducted on the association of *SOST* (gene encodes sclerostin protein) and *LRP5* gene polymorphism and the onset of T2DM in the Indian population. Hence, this study was designed to observe the impact of *SOST* and *LRP5* gene variants in early-onset T2DM in the young population of Uttarakhand.

## Materials and methods

Study design and setting

In this case-control study, consecutive patients between 20 and 40 years with early-onset T2DM who attended Medicine OPD at the All India Institute of Medical Sciences (AIIMS), Rishikesh, were included in the study. The present study was conducted between May 2023 and December 2024. The diagnosis of T2DM was done based on the American Diabetes Association (ADA) criteria. During the screening and recruitment of study participants, individuals who did not develop diabetes mellitus until 40 were recruited under the control group. Patients having type 1 diabetes mellitus and any other chronic diseases, such as thyroid disorder, autoimmune diseases, and malignancy, were excluded from this study.

Sample size

The sample size was calculated using a calculator from Select Statistical Services Limited (Exeter, England), assuming a confidence interval of 95% and an expected odds ratio of 2 with a power of 80%.

Study procedure

The present study commenced after the Institutional Research Ethics Committee of AIIMS Rishikesh issued approval (approval number: AIIMS/IEC/23/168), and informed consent was obtained from all the participants recruited in this study. After doing anthropometric measurements, 5 mL of blood was collected by the skilled phlebotomist, where 3 mL was used for the measurement of biochemical parameters. The lipid profile was measured using the enzymatic method (Beckmann Coulter AU 480, Brea, CA, USA), while intact parathyroid hormone (iPTH) was measured using the chemiluminescent method (Siemens Advia Centaur XP, Tarrytown, NY, USA).

**Figure 1:**
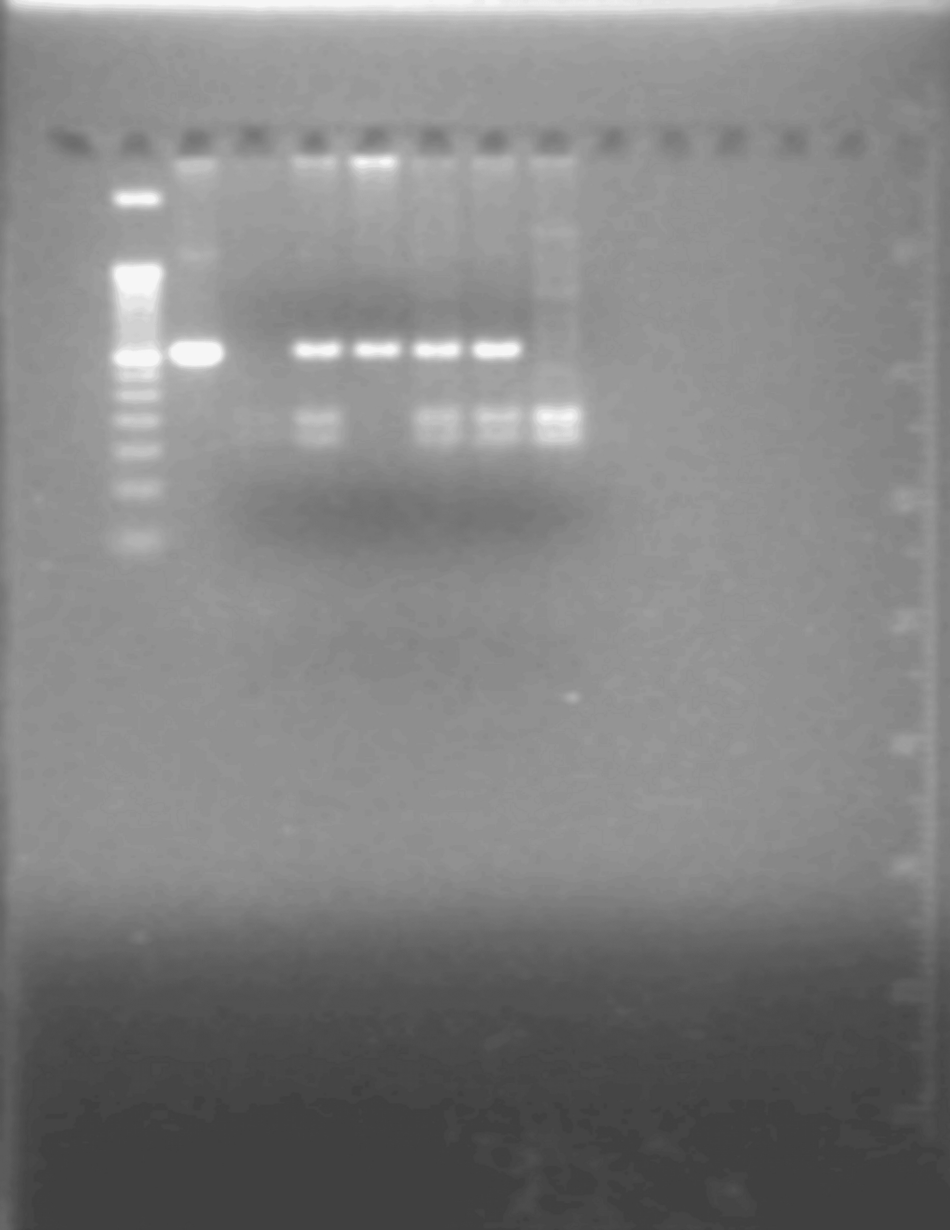
Digested product of the SOST gene on a 3% agarose gel Left to right: lane 1: 50 bp DNA ladder; lane 2: undigested product (365 bp); lanes 3 & 8: homozygous digested (198 & 165 bp); lanes 4, 6, & 7: heterozygous digested (365, 198, 165 bp); lane 5: homozygous undigested (365 bp).

**Figure 2:**
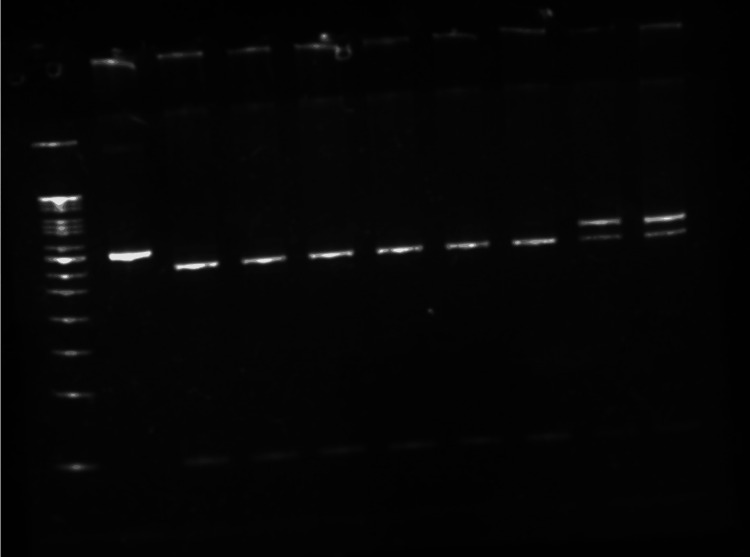
Digested product of the LRP5 gene on native polyacrylamide gel electrophoresis (PAGE) (10%) Left to right: lane 1: 50 bp DNA ladder; lane 2: undigested product (350 bp); lanes 3, 4, 5, 6, 7, & 8: homozygous digested (304 bp & 46 bp); lane 9 & 10: heterozygous digested (350, 304, & 46 bp).

Sclerostin was estimated by the enzyme-linked immunosorbent assay (ELISA) method (Bioassay Technology Laboratory, Shanghai, China). A 2 mL sample of whole blood (EDTA containing) was utilized for genomic DNA extraction by a column-based DNA extraction method using QIAamp kits (QIAGEN, Hilden, Germany), following the manufacturer's protocol. DNA was quantified on a Tecan Multi-Mode Reader (Männedorf, Switzerland) by measuring optical density at 260 nm. The purity of the DNA was assessed by determining the absorbance ratio at 260-280 nm. Genomic DNA integrity was evaluated using agarose gel electrophoresis (1% w/v). Subsequently, the isolated genomic DNA was utilized for PCR amplification of the target gene segment, employing gene-specific primers. In order to identify the genotypes of *SOST* (rs865429G>A) and *LRP5* (rs11228303C>T), polymerase chain reaction-restriction fragment length polymorphism (PCR-RFLP) was employed, followed by agarose gel electrophoresis for larger fragments (Figure 1) and native-PAGE (polyacrylamide gel electrophoresis) for smaller fragments (Figure 2).

The primer sequences and corresponding restriction enzymes are detailed in Tables 1, 2. The amplification was conducted using the Thermocycler (Eppendorf, Germany) in our laboratory. The PCR reaction mixture (20 μL) included genomic DNA (100 ng), PCR master mix 2× (10 μL) consisting of Taq DNA polymerase (0.05 U/μL), MgCl_2_ (4 mM), reaction buffer, dNTPs (0.4 mM each) (Thermo Scientific, MA, USA), and forward and reverse primers (50 pM each) (IDT, Coralville, IA, USA). The thermal cycling conditions began with initial denaturation at 95°C for three minutes, followed by 35 cycles of denaturation at 95°C for 30 seconds, annealing at 60°C for *SOST* and 59°C for *LRP5* for 30 seconds. Extension temperatures were 72°C with durations of 20 seconds. A final extension step at 72°C for five minutes was applied across all genes.

**Table 1:** Primer sequences of SOST and LRP5 gene variants Designed using Primer 3.0 software (San Francisco, CA, USA) synthesized from IDT (Coralville, IA, USA), along with annealing temperature and amplicon size for each variant

Gene	Primer	Product Sizes	Annealing Temperature
SOST	F: GAGGTGAACCCCCAGCTCGAAG	365	60°C
	R: GCAAGGTTGGGACTGGGGTGG		
LRP5	F: CTACCCAAATCCTATAAA	350	59°C
	R: GGGCTATGAGCTAGTTAAG		

**Table 2:** Restriction enzymes and the pattern of digestion, along with their base pair sizes for different polymorphic variants of the SOST and LRP5 genes

Name of Gene	Genotypic Variant	Restriction Enzyme	Homozygous Undigested	Homozygous Digested	Heterozygous
SOST	rs865429 G>A	NCoI	365	198, 165	365, 198, 165
LRP5	rs11228303C>T	HinfI	350	304, 46	350, 304, 46

The resulting PCR products were then subjected to restriction enzyme digestion according to the specified enzymes in Table 2. This was followed by electrophoresis on agarose gels for larger fragments and native-PAGE for smaller ones, facilitating genotype determination.

Statistical analysis

Data were analyzed using IBM SPSS Statistics for Windows, Version 23 (Released 2015; IBM Corp., Armonk, New York). Categorical data were expressed as percentages and continuous variables as means ± SD or medians (interquartile range) depending on the normality distribution of data. Comparisons between variables were conducted using the Mann-Whitney U test/Student's t-test and analysis of variance/Kruskal-Wallis rank test. Observed and expected genotype frequencies were measured using the chi-square test for Hardy-Weinberg equilibrium analysis. The odds ratio, along with a 95% confidence interval, was calculated. A p-value less than 0.05 was considered significant.

## Results

This study recruited 114 early-onset T2DM cases and 115 sex-matched control subjects. Table 3 depicts the demographic and biochemical features of the cases and controls.

**Table 3:** Demographic and biochemical parameters of all study participants

Parameter	Cases (114)	Control (115)	p-value
Gender (F/M)	48/66	49/66	
BMI (kg/m^2^)	21.95±10.09	24.09±7.57	0.072
Adiposity index (%)	35.29±17.34	39.25±14.94	0.066
Waist-hip ratio	0.88±.25	1.08±1.21	0.099
SBP (mmHg)	132.95±20.06	136.80±21.25	0.213
HbA1C (%)	8.20±3.17	8.35±6.14	0.844
Total cholesterol (mg/dL)	196.93±51.46	193.79±55.60	0.668
TG (mg/dL)	170 (151)	144 (132)	0.989
LDL (mg/dL)	112.58±38.90	121.71±50.29	0.139
Calcium (mg/dL)	9.72±1.26	10.07±3.65	0.391
iPTH (pg/mL)	45 (43)	52 (34)	0.440
Sclerostin (pmol/L)	46.20 (16.81)	49.36 (17.82)	0.486

The frequency distribution of the *SOST* rs865429 G>A and *LRP5* rs11228303C>T genotypes adhered to the Hardy-Weinberg equilibrium (p>0.05). In early-onset diabetes cases, the genotype distribution is rs865429.SOST: AA (73.7%, n=84), AG (24.6%, n=28), and GG (1.8%, n=2), while controls showed frequencies of AA (68.7%, n=79), AG (30.4%, n=35), and GG (0.9%, n=1). Allele frequencies for the A allele were higher in both cases (85.96%, n=196) and controls (83.91%, n=193) compared to the G allele, which was less frequent in cases (14.03%, n=32) and controls (16.08%, n=37), mirroring the genotype distribution. The GG genotype showed a higher odds ratio (OR=2.035) compared to the AA and AG genotypes, but this finding lacks statistical significance (p=0.564) due to the small sample size (Table 4). The association between the genotype and clinicopathological association is presented in Table 5. In the cases group, the GG genotypes showed significantly higher LDL levels compared to the AA and AG genotypes (p=0.027). Moreover, this GG genotype shows higher HbA1c, total cholesterol, and triglyceride levels compared to other genotypes, although they are not statistically significant. Moreover, there is a nonsignificant higher level of calcium, iPTH, and sclerostin in the GG genotype compared to other genotypes, suggesting a possible interaction between the *SOST* variant and bone metabolism.

**Table 4:** Genotype and allele frequency of rs865429 G>A of SOST gene

SOST Frequency		Cases	Control	Odds Ratio	95% CI	p-value
Genotype	AA	84 (73.7%)	79 (68.7%)	1.275	0.718 to 2.264	0.405
	AG	28 (24.6%)	35 (30.4%)	0.744	0.4155 to 1.332	0.320
	GG	2 (1.8%)	1 (0.9%)	2.035	0.182 to 22.770	0.564
Allele	A	196 (85.96%)	193 (83.91%)	1.174	0.702 to 1.961	0.539
	G	32 (14.03%)	37 (16.08%)	0.851	0.509 to 1.422	0.539

**Table 5:** Associations between SOST genotype and clinicopathological variables BMI: body mass index; HbA1c: hemoglobin A1C; TG: triglyceride; LDL: low-density lipoprotein; iPTH: intact parathyroid hormone

Total study participants				
Parameters	AA (162)	AG (63)	GG (3)	p-value
BMI	22.66±9.25	23.207±8.679	31.633±4.398	0.229
Adiposity index	37.75±16.42	36.090±17.177	49.166±9.963	0.374
Waist-hip ratio	0.89±0.25	1.196±.626	0.973±.085	0.266
HbA1c	7.79±2.80	9.380±3.076	10.800±3.252	0.143
Total cholesterol	191.60±56.11	201.600±48.384	222.333±41.29	0.324
TG	178.56±55.22	200.852±93.281	213.666±94.543	0.384
LDL	105.51±48.58	122.882±58.986	124.667±10.692	0.027
Calcium	9.77± 4.12	10.229± 4.183	10.000± 3.121	0.600
iPTH	50.30 (40)	46.8 (37)	73.5 (42.45)	0.572
Sclerostin	46.11 (21.31)	48.461 (16.34)	53.734 ± 4.348	0.757
In Cases				
Parameters	AA (84)	AG (28)	GG (2)	p-value
BMI	21.32± 10.47	22.56±9.91	29.10±0.42	0.514
Adiposity index	34.97±17.89	35.67±16.39	43.55±3.04	0.784
Waist-hip ratio	0.87±0.28	0.88±0.25	1±0.09	0.785
HbA1c	5.30±4.72	6.04±4.54	4.25±6.01	0.722
Total cholesterol	178.85±76.30	187.07±63.42	208.00±46.67	0.766
TG	175.64±138.13	179.18±95.17	157±50.91	0.970
LDL	99.68±50.41	114.67±41.43	118.50±0.71	0.332
Calcium	7.73±4.10	6.65±4.72	9.60±0.57	0.394
iPTH	52.75±28.30	45.93±19.11	73.30 (44.55)	0.525
Sclerostin	45.09 (22.91)	47.57 (19.85)	49.97 ± 7.53	0.730

In the total participants, the *LRP5* CC genotype was predominant in both the cases and control groups, with a higher frequency observed in the control group. The allelic frequencies show a similar pattern, with allele C being more prevalent than allele T in both groups (Table 6).

**Table 6:** Genotype and allele frequency of the LRP5 gene rs11228303C>T

*LRP5* Frequency		Cases	Control	Odds Ratio	95% CI	p-value
Genotype	CC	95 (82.6%)	101 (87.8%)	0.693	0.329 to 1.459	0.334
	CT	179 (14.8%)	13 (11.3%)	1.375	0.634 to 2.98	0.410
	TT	2 (1.7%)	1 (0.9%)	2.035	0.182 to 22.77	0.563
Allele	C	207 (90.78%)	215 (93.47%)	0.687	0.3451 to 1.370	0.287
	T	21 (9.21%)	15 (6.52%)	1.454	0.7297 to 2.897	0.287

The association between the LRP5 genotype and biochemical parameters is shown in Table 7. The CT genotype shows significantly (p=0.018) higher HbA1c levels (11.22±2.04) compared to the CC (7.88±2.84) and TT (6.15±0.07) genotypes. The CT genotype also showed elevated levels of triglycerides and calcium, whereas the TT genotype showed increased levels of total cholesterol and LDL. Interestingly, in early-onset diabetes cases, the TT genotype showed elevated levels of all the clinicopathological parameters compared to the CC and CT genotypes (Table 7).

**Table 7:** Associations between LRP5 rs11228303C>T genotype and clinicopathological variables BMI: body mass index; HbA1c: hemoglobin A1C; TG: triglyceride; LDL: low-density lipoprotein

Total study participants				
Parameters	CC (196)	CT (30)	TT (3)	p-value
BMI	23.33±8.76	20.64±11.14	22.37±2.61	0.319
Adiposity index	38.10±15.69	33.06±21.77	39.23±4.40	0.295
Waist-hip ratio	0.95±0.69	1.18±1.69	0.95±0.03	0.418
HbA1c	7.88±2.84	11.22± 2.04	6.15±0.07	0.018
Total cholesterol	195.59±54.77	184.68±48.09	226±30.05	0.376
TG	183.45±99.59	207.40±82.09	137.66±34.67	0.450
LDL	110.02±45.93	109.07±81.98	134.66±20.03	0.710
Calcium	9.73±0.98	11.05±7.69	9.83±0.25	0.102
In Cases				
Parameters	CC (95)	CT (17)	TT (2)	p-value
BMI	21.56±10.04	22.33±12.11	26.60±2.97	0.769
Adiposity index	34.69±17.01	37.51±20.09	45.20±4.95	0.597
Waist-hip ratio	0.88±0.26	0.84±0.32	0.95±0.01	0.776
HbA1c	5.70±4.61	4.21±4.85	5.20±7.35	0.484
Total cholesterol	180.29±71.48	181.26±83.20	234±11.31	0.590
TG	179.95±134.23	153.93±89.34	186.50±23.33	0.738
LDL	102.60±47.38	105.85±55.32	137±9.90	0.599
Calcium	7.57±4.22	6.84±4.58	9.85±0.3.53	0.596

## Discussion

This study explored the associations between the *SOST* (rs865429 G>A) and *LRP5* (rs11228303C>T) genotypes and various biochemical parameters in early-onset T2DM cases and matched controls. Our analysis indicated trends in genotype distributions and biochemical parameter associations that, while not always statistically significant, may shed light on potential genetic contributions to metabolic dysregulation in early diabetes.

The genotype distribution for the *SOST* rs865429 G>A variant complied with the Hardy-Weinberg equilibrium in both cases and controls, indicating that the observed frequencies were comparable with the expected genetic proportions in a stable population. In early-onset T2DM cases, the CC genotype was most prevalent, with frequencies like those seen in controls. Despite an increased odds ratio (OR=2.035) for the GG genotype relative to AA and AG, the non-significant association (p=0.564) could be the result of our small sample size. Larger studies might confirm whether the increased GG genotype frequency among cases contributes to the true genetic predisposition for early-onset T2DM.

The potential link between the *SOST* rs865429 GG genotype and higher HbA1c, cholesterol, and LDL levels mirrors findings in related research, where *SOST* had been implicated in metabolic dysregulation [7]. Elevated HbA1c in the GG genotypes may reflect the broader systemic effects of this variant on glucose metabolism. Other studies have shown that *SOST* variants might indirectly affect glucose levels through altered bone-derived signaling, as bone and glucose metabolism are increasingly recognized as interconnected. Additionally, previous research has shown that *SOST* may induce altered lipid metabolism via insulin resistance and induction of proinflammatory cytokines, which could partly explain the observed elevated HbA1c levels in the GG genotype in our study [8].

An in vitro study conducted by Sidgwick et al. in 2024 documented that *SOST* induces metabolic disorder via downregulation of the RAGE/ERK/CREB pathway, leading to attenuation of the expression of genes involved in lipid metabolism [9]. This study's observation of higher LDL levels in GG carriers aligns with these findings and supports that a fraction of early-onset diabetic individuals might be prone to developing cardiovascular disorders, which is in agreement with a previous study conducted by Ali and Nima (2021), although further research with larger samples would be necessary to establish a statistically significant relationship [10].

Silva and Bilezikian (2015) elucidated that PTH exerts dual (complex) effects on the skeleton [7]. Intermittent controlled exposure to PTH reduces sclerostin expression, promoting osteoblastic activity, which in turn causes bone formation. On the other hand, continuous exposure to high parathormone facilitates bone resorption via enhanced RANKL/OPG ratios. Calcium levels regulate iPTH secretion, which in turn modulates bone metabolism through sclerostin production. This tightly regulated loop maintains calcium homeostasis. The increased iPTH level, in addition to non-significantly elevated calcium and sclerostin levels in the GG genotype, might validate the role of *SOST* variants in the modulation of calcium and bone metabolism in the context of early-onset T2DM. Higher levels of sclerostin in the GG genotype might explain the increased susceptibility to the development of osteoporosis and bone fracture in a subfraction of diabetic individuals, which agrees with a previous study conducted by Deveci et al. in 2018 [11]. The classical picture of tight regulation of three parameters could not be demonstrated since this study dealt with mostly early-onset diabetes cases.

The *LRP5* genotype distribution also followed the Hardy-Weinberg equilibrium. Interestingly, the CC genotype was predominant in both cases and controls, and allele frequency analysis mirrored the genotype distribution pattern. However, the nonsignificant association of the broadly prevalent genotype could not confirm whether this genotype had a potentially protective or predisposing nature for early-onset T2DM, and thus, further exploration is warranted.

Within the total participant group, the CT genotype documented significantly increased HbA1c levels compared to the CC and TT genotypes (p=0.018), suggesting an association between this genotype and poorer glycemic control. This is in concordance with a previous study conducted by Fujino et al. (2003), who documented the crucial role of *LRP5* in bone metabolism and glucose homeostasis because of its involvement in the Wnt/β-catenin signaling pathway [6]. This relationship was accompanied by higher triglyceride and calcium levels in the CT variant, while the TT genotype was associated with higher LDL and total cholesterol levels. Interestingly, among early-onset diabetes cases, the TT genotype showed higher levels across all clinicopathological parameters, which indicates that the TT genotype might contribute to a broader pattern of metabolic dysregulation in early-onset diabetes cases. On the other hand, Zhang et al. could not find any direct association between certain *LRP5* SNPs and T2DM incidence, possibly due to population differences since polymorphism varies with race and ethnicity [12].

Limitations and future directions

This is the first study to observe the association between the *SOST* and *LRP5* genotypes and early-onset T2DM. However, this study has some limitations. The modest sample size might have limited the detection of associations involving less frequent genotypes. Despite notable trends, the lack of significance warrants the need for larger cohorts to validate these preliminary associations. Additionally, this study did not account for environmental factors, lifestyle, or medications that could also affect biochemical profiles. Moreover, a single-centered study might limit its generalizability.

Future studies could explore these genotypes' role in diverse populations and assess interactions between genetic and environmental factors in early-onset T2DM. Moreover, investigating additional SNPs within the *SOST* and *LRP5* genes might provide insight into a more comprehensive understanding of these loci in diabetes risk among the young population.

## Conclusions

In conclusion, elevated levels of markers of glucose homeostasis, lipid metabolism, and bone metabolism among GG and TT genotypes suggest that the *SOST* rs865429 and *LRP5* rs11228303 variants may play a role in diabetes pathophysiology and predispose a subset of individuals to early-onset T2DM and its complications. Our findings underscore the impact of underlying genetic predispositions toward early-onset T2DM, which requires tailored approaches both clinically and therapeutically based upon personalized risk profiles derived from comprehensive genotypic assessments. However, larger multicenter studies are needed to validate these preliminary findings.
